# Maturation and integration of adult born hippocampal neurons: signal convergence onto small Rho GTPases

**DOI:** 10.3389/fnsyn.2013.00004

**Published:** 2013-08-20

**Authors:** Krishna C. Vadodaria, Sebastian Jessberger

**Affiliations:** ^1^Brain Research Institute, University of ZurichZurich, Switzerland; ^2^Neuroscience Center Zurich, University of Zurich and ETH ZurichZurich, Switzerland

**Keywords:** neurogenesis, Rac1, Cdc42, RhoA, synaptic integration, spine growth, dendrite, *in vivo*

## Abstract

Adult neurogenesis, restricted to specific regions in the mammalian brain, represents one of the most interesting forms of plasticity in the mature nervous system. Adult-born hippocampal neurons play important roles in certain forms of learning and memory, and altered hippocampal neurogenesis has been associated with a number of neuropsychiatric diseases such as major depression and epilepsy. Newborn neurons go through distinct developmental steps, from a dividing neurogenic precursor to a synaptically integrated mature neuron. Previous studies have uncovered several molecular signaling pathways involved in distinct steps of this maturational process. In this context, the small Rho GTPases, Cdc42, Rac1, and RhoA have recently been shown to regulate the morphological and synaptic maturation of adult-born dentate granule cells *in vivo*. Distinct upstream regulators, including growth factors that modulate maturation and integration of newborn neurons have been shown to also recruit the small Rho GTPases. Here we review recent findings and highlight the possibility that small Rho GTPases may act as central assimilators, downstream of critical input onto adult-born hippocampal neurons contributing to their maturation and integration into the existing dentate gyrus (DG) circuitry.

## Introduction

Throughout lifespan new neurons are continuously born in the mammalian hippocampus. It is now widely accepted that the process of adult neurogenesis is not merely a remnant of embryonic development, but a highly responsive and regulated process that appears to be critically involved in hippocampus-dependent behavior in health and disease (Sahay and Hen, 2007; Zhao et al., 2008; Danzer, 2012). The number of newborn neurons in the adult hippocampus is not static but strongly influenced by many positive and negative stimuli that influence the neurogenic process at distinct developmental stages. Positive extrinsic regulators include physical exercise, environmental enrichment, antidepressants, and learning, whereas stress and aging negatively regulate the number of newly generated neurons (Ma et al., 2009). These stimuli are thought to impact adult neurogenesis via a number of regulatory pathways including growth factors, neurotransmitters, developmental signaling molecules, and hormones. Inspired by previous studies in the context of embryonic neurogenesis, a number of cellular and molecular mechanisms, involved in the control of neural stem/progenitor cells (NSPC) activity and subsequent integration of newborn granule cells within the adult hippocampus, have been identified (Ming and Song, 2011). Even though it has been demonstrated that the neurogenic process in the adult hippocampus shares many properties with embryonic neurogenesis, it principally differs from embryonic neurogenesis in that, NSPCs and maturing neurons are present in an entirely different (adult) environment and must integrate into a preexisting circuit, presumably in the absence of large amounts of developmental guidance cues (Conover and Notti, 2008). Thus, understanding the molecular underpinnings of how adult-born neurons integrate into the dentate gyrus (DG) circuitry requires experiments that analyze the cellular mechanisms and signaling pathways in their endogenous adult niche.

Recently, a few reports demonstrate stage-specific roles for small Rho GTPases, Cdc42, Rac1, and RhoA in adult hippocampal neurogenesis *in vivo*. These studies suggest an important and potentially central role for the small Rho GTPases in the maturation and integration of newborn neurons in the adult hippocampus. In this mini-review, we discuss the main findings of recent *in vivo* studies and then focus on discussing how known upstream intrinsic regulators (neurotrophins, neurotransmitters, developmental signaling molecules, and intermediate signaling molecules) maybe recruiting Rho GTPases for mediating their effects on neuronal maturation in adulthood. We begin by describing the maturation process of adult born hippocampal neurons and give a broad overview of neuronal Rho GTPase signaling, followed by a discussion of evidence showing how important regulators of neuronal maturation may be modulating small Rho GTPases as downstream effectors. We conclude with hypotheses on mechanisms for signal convergence and a brief discussion of how Rho GTPases may act to assimilate multiple upstream signals to decisively influence cell cytoskeleton and neuronal cytoarchitecture. For a more detailed discussion on Rho GTPase signaling in neurons, please refer to other reviews (Auer et al., 2011; Govek et al., 2011; Chen et al., 2012).

## Maturation of adult-born hippocampal neurons

**Figure 1:**
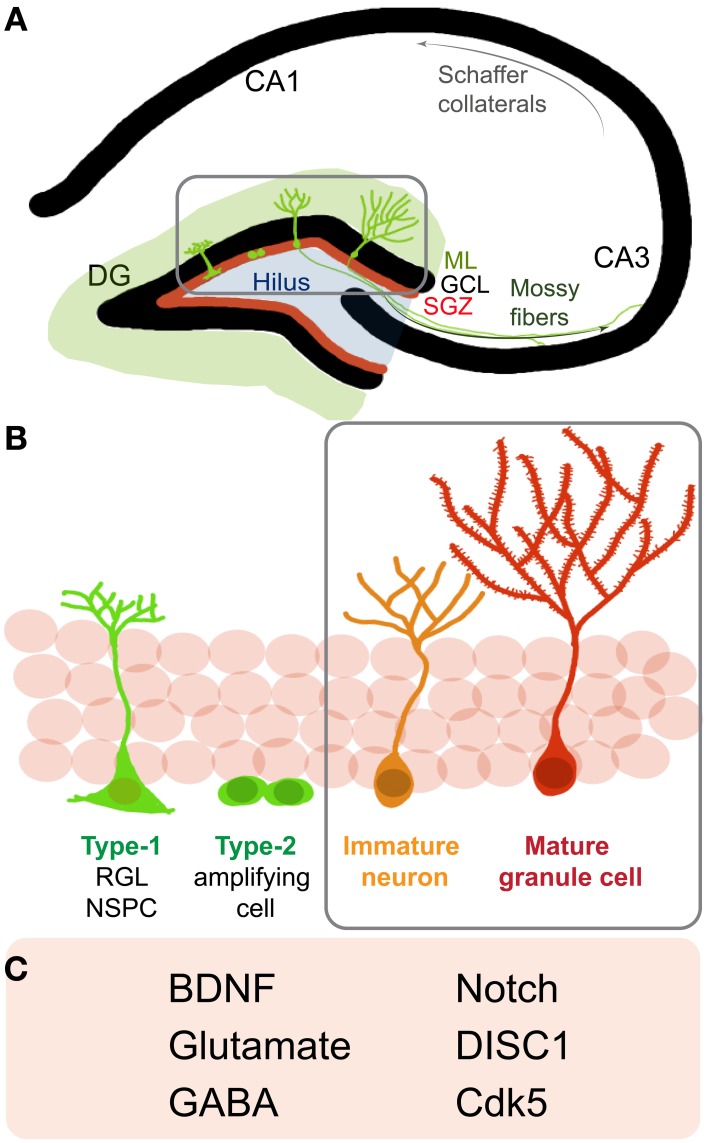
**Neurogenesis in the adult rodent hippocampus (A) Schematic of a coronal section of the hippocampus: cornu ammonis (CA) regions and the dentate gyrus (DG).** Depicted are neural stem/progenitor cells (NSPCs) (green cells) residing in the subgranular zone (SGZ, red line) at the border of the granule cell layer (GCL) and molecular layer (ML). (Gray box) NSPCs divide, mature and go through the different stages of development and send out dendrites into the molecular layer and axons to the CA3 region via the mossy fiber pathway. **(B)** A closer look at neurogenesis reveals that adult NSPCs go through distinct stages of maturation where Type-1, radial glia-like stem cells give rise to Type2 transit amplifying cells, which divide to generate immature neurons that start developing characteristic DG granule cell morphology and finally mature and integrate into the DG circuitry as mature granule cells. **(C)** Listed are some notable regulators of later stages **(B**, gray box**)** of newborn neuron maturation and integration: brain derived neurotrophic factor (BDNF), gamma-Aminobutyric acid (GABA), Disrupted-in-Schizophrenia 1 (DISC1), cyclin-dependent kinase 5 (Cdk5).

Adult hippocampal NSPCs go through distinct stages of maturation on their way to becoming fully mature newborn granule cells (Figure 1). It is currently assumed that radial glia-like NSPCs (type 1 cells) give rise to non-radial glia-like transit amplifying precursors (type 2 cells) that divide and generate immature neurons. These immature neurons grow an apical dendrite towards the molecular layer and send axonal processes to their target area, the CA3, several days after they are born (Zhao et al., 2006) (Figure 1A). During this period of maturation, newborn cells display distinct electrical properties, including gamma-aminobutyric acid (GABA)-induced depolarization, contributing to their survival and functional integration into the adult hippocampal circuitry (Ge et al., 2006) (Figures 1B,C). As these neurons further mature, they start receiving excitatory synaptic input, develop dendritic spines, and display extensive dendritic arborization. Adult-generated young granule cells display hyperexcitability as compared to granule cells generated during development (Wang et al., 2000; Schmidt-Hieber et al., 2004; Marin-Burgin et al., 2012). This property conceivably enables cohorts of newborn neurons to encode time (temporal context) within memory and allows the separation of patterns that are closely related, spatially or temporally (Aimone et al., 2010; Deng et al., 2013). In the rodent DG, it takes ~8 weeks for adult-born granule cells to become nearly indistinguishable from developmentally-generated granule cells (Laplagne et al., 2006). Notably, each successive developmental stage is sensitive to a number of extrinsic and intrinsic regulators (Zhao et al., 2008; Ming and Song, 2011).

Molecular regulators of maturation and integration of adult-born neurons include neurotrophins such as brain derived neurotrophic factor (BDNF), neurotransmitters such as GABA and glutamate, and signaling molecules like Disrupted-in-Schizophrenia 1 (DISC1) (Figure 1C). These regulators recruit diverse downstream pathways to finally influence distinct aspects of neuronal maturation such as migration, dendritic arborization, spine maturation and synaptic integration of newborn hippocampal neurons (Jagasia et al., 2006; Hagg, 2007). Thus, one may speculate that these molecules and their downstream effectors may partially impinge on some common signaling pathways to influence neuronal maturation. Several lines of evidence indicate that the small Rho GTPases are important and central regulators of cell cytoarchitecture in different cell types and play important roles in modulating cell migration, neurite outgrowth, survival, as well as synapse formation in neurons (Govek et al., 2005; Newey et al., 2005; Watabe-Uchida et al., 2006; Svitkina et al., 2010). Hereon, we focus on how the aforementioned regulators interact with and influence small Rho GTPase signaling to possibly modulate neuronal integration in the adult hippocampus.

## Small Rho GTPase signaling

**Figure 2:**
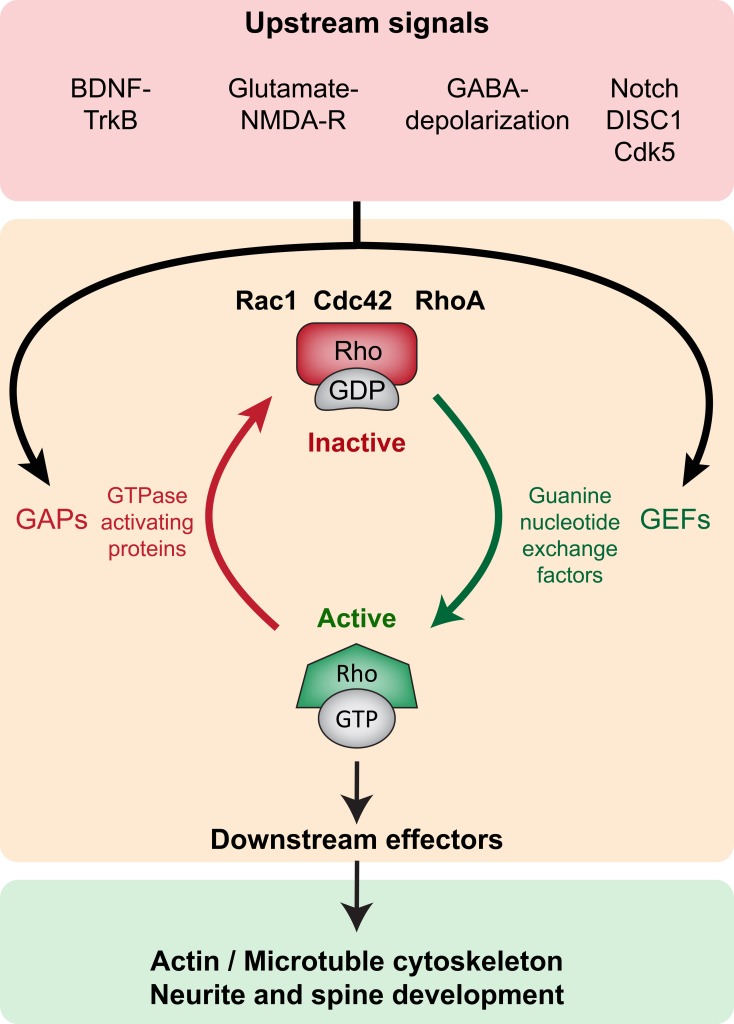
**Small Rho GTPase signaling in neurite and spine maturation (Top panel, red).** Upstream signaling such as BDNF-TrkB, Glutamate-NMDAR, GABA-induced depolarization, Notch, DISC1 and Cdk5 modulate small Rho GTPase activity. (Middle panel, orange) Upstream signals modulate GEFs and GAPs thereby regulating the activity of small Rho GTPases Rac1, Cdc42, and RhoA. GEFs promote activation (GTP-bound conformation) and GAPs promote inactivation (GDP-bound conformation). **(Bottom panel, green)** In the active state, small Rho GTPases bind to several downstream effectors exerting influence on local actin and microtubule networks, thereby influencing neurite and spine growth.

Rho GTPases are part of the larger Ras superfamily of monomeric GTPases. These small GTPases are thought to act as binary molecular switches, transducing upstream signals to downstream effectors by alternating between the “active” GTP-bound and the “inactive” GDP-bound state (Schmitz et al., 2000) (Figure 2). Conversion from one state to the other is tightly regulated by guanine nucleotide exchange factors (GEFs), the GTPases activating proteins (GAPs) and the guanine nucleotide dissociation inhibitors (GDIs). Small Rho GTPase activity states are regulated by the activating GEFs, which promote exchange of GDP for GTP; the inactivating GAPs (GTPases activating proteins), which enhance the intrinsic capacity of the small GTPases for hydrolyzing GTP to GDP; and the inactivating GDIs that prevent dissociation of GDP from the GTPases (as there are usually higher levels of intracellular GTP than GDP) (Jaffe and Hall, 2005). GDIs further inhibit GTPase activity by sequestering them in the cytoplasm, as opposed to the cell membrane where they functionally localize due to posttranslational lipid modifications such as prenylation and palmitoylation (Kang et al., 2008; Samuel and Hynds, 2010; Navarro-Lerida et al., 2012). GEFs, GAPs, and GDIs are specific to different Rho GTPases, and their expression and subcellular localization is crucial to the spatial and temporal regulation of Rho GTPase activity. Activated GTPases (GTP-bound) bind to several downstream effectors that directly modify the actin and microtubule cytoskeletal network, thereby influencing a variety of processes such as cell growth, survival, proliferation, membrane trafficking, transcriptional activation, adhesion, mechanosensation, and migration (Van Aelst and D'Souza-Schorey, 1997; Schmitz et al., 2000; Govek et al., 2005; Watabe-Uchida et al., 2006; Govek et al., 2011; Keung et al., 2011) (Figure 2).

Cdc42 (cell division cycle 42), Rac1 (ras-related C3 botulinum toxin substrate 1) and RhoA (ras homolog family member A) are the best-studied members of the small Rho GTPase family, especially in the neuronal context. Despite early indications of important roles of the Rho GTPases in neuronal maturation *in vitro*, there are far fewer reports examining their role in the mammalian CNS *in vivo* (Heasman and Ridley, 2008). This is due to the fact that straight knockouts of Cdc42 and Rac1 are lethal during embryogenesis, with death occurring by E9.5 (Sugihara et al., 1998; Chen et al., 2000). Newer studies, using region-specific conditional deletion of the small GTPases during development, demonstrate diverse roles for Cdc42, Rac1 and RhoA in embryonic neurogenesis and neuronal maturation (Luo et al., 1996; Cappello et al., 2006, 2012; Chen et al., 2007; De Curtis, 2008; Fuchs et al., 2009; Haditsch et al., 2009; Leone et al., 2010; Vaghi et al., 2012). Only very recently have studies begun investigating the role of the small GTPases in the adult CNS *in vivo*. These studies show roles for Rho GTPase signaling in SVZ/OB (Ding et al., 2010; Leong et al., 2011) and hippocampal neurogenesis (Keung et al., 2011; Christie et al., 2013; Vadodaria et al., 2013). Here, we focus on recent findings demonstrating specific roles for Rho GTPases in adult hippocampal neurogenesis.

In their study, using retrovirus mediated overexpression of constitutively active (CA, GTP-bound) and dominant negative (DN, GDP-bound) forms of RhoA in adult hippocampal neurons *in vivo*, Keung et al. found an inverse relationship between RhoA activity and the percentage of cells differentiating into neurons (Keung et al., 2011). However, it is possible that this effect on neuronal differentiation was due to an effect on survival of newborns and not differentiation per se, as shown recently by Christie et al. Here, the authors blocked RhoA signaling pharmacologically *in vivo* and observed enhanced spatial memory in the Y-maze along with an increase in newborn neuron survival, and no differences in NSPC proliferation or differentiation (Christie et al., 2013). These findings suggest that RhoA may have a “negative” role in neuronal survival and maturation. However, due to off-target effects of DN/CA forms and pharmacological inhibitors, additional studies genetically knocking down RhoA *in vivo*, would help clarify its precise role in adult hippocampal neurogenesis. In our study, using both genetic deletion *in vivo* and DN-form overexpression, we found stage-specific roles for Cdc42 and Rac1 in adult hippocampal neurogenesis. Cdc42 activity was found to be important for normal levels of proliferation, as well as dendritic and spine maturation. In contrast, Rac1 was found to be specifically important for later stages of dendritic and spine maturation (Vadodaria et al., 2013). Collectively, results from the above mentioned studies suggest that Cdc42 and Rac1 are involved in proliferation, and dendritic and spine maturation, whereas RhoA may inhibit survival and growth of newborn neurons. In addition to playing a role in adult hippocampal neurogenesis, Rac1 and its epigenetic regulation was found to regulate synaptic plasticity and spine remodeling in the adult nucleus accumbens, where it is functionally involved in addiction (Dietz et al., 2012).

Taken together, these findings hint at stage-specific roles of the Rho GTPases in adult hippocampal neurogenesis. This may be due to differential recruitment of the Rho GTPases by upstream factors during different stages of neuronal maturation. We next discuss how upstream signaling molecules may recruit Cdc42, Rac1, and RhoA, for mediating their effects on maturation and integration of newborn neurons in the adult hippocampus.

## Regulators of neuronal maturation: ties with small Rho GTPase signaling

### Neurotrophin signaling: BDNF

BDNF is among the most well studied neurotrophins and has been shown to have a specific role in maturation and integration of newborn neurons in the adult hippocampus (Schmidt and Duman, 2007). Conditional mutants lacking BDNF exhibit a specific defect in newborn neuron maturation, but not in proliferation, cell fate specification, or survival. In particular, loss of BDNF leads to an accumulation of immature neurons with significantly shorter dendrites (Chan et al., 2008). Although BDNF binds multiple Trk receptors as well as the non-specific p75 neurotrophin receptor (NTR), it appears that the effects of BDNF deletion on neuronal morphology are likely mediated by its main receptor, TrkB (tropomyosin receptor kinase B). Conditional deletion of TrkB in adult NSPCs leads to a specific reduction in dendritic arborization and spine density (Bergami et al., 2008). Further, stimuli such as stress, exercise, and antidepressants that are known to regulate adult neurogenesis (and hippocampus-dependent behavior) act via regulation of BDNF signaling (Duman and Monteggia, 2006). These studies suggest an indispensible role for BDNF-TrkB signaling in the maturation and integration of newborn neurons in the adult hippocampus.

The above-mentioned *in vivo* studies shed light on BDNF's role in hippocampal neuron maturation, and the molecular mechanisms downstream of BDNF-TrkB signaling have been explored mainly using primary cultures *in vitro*. Several studies have established Rho GTPases to be downstream of TrkB activation. BDNF-TrkB signaling has been shown to specifically modulate cell morphology of hippocampal neurons *in vitro*, via activation of GEF Tiam1 and Rac1, and through Cdk5 dependent activation of Cdc42 (Miyamoto et al., 2006; Cheung et al., 2007). In addition to directly targeting Cdc42 and Rac1, TrkB also regulates dendritic morphogenesis by activating geranylgeranyltransferase-I, which performs lipid modifications (prenylation) of the Rho GTPases, enabling their localization at the cell membrane (Zhou et al., 2008). This may be particularly relevant as recruitment of Rac1 to lipid rafts in hippocampal neurons is crucial to the biological activity of neurotrophins like, nerve growth factor (Fujitani et al., 2005). These results suggest that BDNF-TrkB signaling recruits and activates the positive regulators of neurite outgrowth Cdc42 and Rac1. BDNF and its protein precursor proBDNF bind to the non-specific neurotrophin receptor p75 (p75NTR), which has been shown to negatively impact cell survival and neurite outgrowth (Nykjaer et al., 2005; Blochl and Blochl, 2007). Interestingly, proBDNF-p75NTR signaling promotes growth cone collapse via downstream activation of RhoA (Sun et al., 2012). This fits in well with the notion that RhoA generally promotes growth cone collapse, whereas Cdc42 and Rac1 promote neurite growth and stabilization (Huang and Reichardt, 2003). Taken together, these studies indicate a strong link between BDNF-TrkB signaling and the regulation of small Rho GTPases for modulation of neurite growth dynamics. Given this, it is possible to hypothesize that Cdc42, Rac1 and RhoA may be recruited in a similar fashion downstream of BDNF for maturation of adult-born hippocampal neurons *in vivo*.

### Neurotransmitter signaling: glutamate and GABA

Neuronal activity is central to the development of neurons, and neurotransmitters have been shown to regulate distinct stages of maturation of adult-born neurons (Vaidya et al., 2007). Glutamate and GABA primarily regulate excitation and inhibition in the adult nervous system, respectively, and have been shown to play important roles in the maturation of adult-born hippocampal neurons. Previous studies using pharmacological tools have demonstrated the importance of activity in neuronal maturation, and a key role for N-Methyl-D-aspartic acid receptor (NMDAR)-driven excitation in neuronal maturation has since long been proposed (Brewer and Cotman, 1989; McKinney et al., 1999; Richards et al., 2005; Nacher and McEwen, 2006). More recent studies have capitalized on the development of finer molecular biology tools to examine the stage-specific roles of glutamate and GABA in distinct stages of neuron maturation. Tashiro et al. showed that NMDAR-driven excitation is critical for survival and integration of newborn neurons using retrovirus-mediated single-cell knockout of NMDAR in adult-born hippocampal neurons, (Tashiro et al., 2006). Not only is glutamatergic excitation important but also GABA-induced depolarization is crucial during early stages of newborn neuron maturation. Early excitatory GABAergic input critically regulates the survival and maturation of adult-born hippocampal neurons (Ge et al., 2006, 2008). The effects of early GABAergic input on newborn neurons are likely mediated via cAMP response element-binding protein (CREB) signaling (Jagasia et al., 2009). Furthermore, recent studies have revealed which GABA receptors and subunits are involved in mediating the effects of GABA on the newborn progenitors. Adult-born hippocampal neurons of mutant mice lacking the α 4-subunit-containing GABA_A_Rs and α2-subunit-containing GABA_A_Rs show defects in dendritic arborization, and cell migration in a temporally distinct manner (Duveau et al., 2011). This differs from the role of γ2-subunit-containing GABA_A_Rs, which regulate early stages of stem cell division, as NSPCs lacking the γ2-subunit exit quiescence and exhibit increased symmetrical divisions (Song et al., 2012). Collectively, these results indicate that glutamatergic and GABAergic input play important roles in the maturation of newborn hippocampal neurons *in vivo*.

The small Rho GTPases appear to be regulated by both glutamatergic and GABAergic neurotransmission via specific GEFs. Although glutamate initially and transiently activates RhoA (Jeon et al., 2002), longer activation of NMDAR leads to robust activation of the Rac1 GEF, Tiam1, specifically in dendrites and spines of hippocampal neurons *in vitro* (Tolias et al., 2005). This suggests that glutamate may transiently upregulate RhoA activity via AMPA receptors initially, whereas, longer or coincident excitation via NMDARs likely recruits Rac1 for mediating its positive influence on neurite growth and spine motility (Tashiro and Yuste, 2008). This is significant given that localized activation of the Rho GTPases has been directly linked to plasticity at single dendritic spines (Murakoshi et al., 2011; Yasuda and Murakoshi, 2011). GABAergic signaling has also been linked to the Rho GTPases Cdc42 and Rac1. Meyer et al. show that Rac1 activity is required for optimal GABA_A_ receptor activity, as a loss of Rac1 activity reduced GABA_A_ receptor activity, possibly through loss of receptor clustering and recycling (Meyer et al., 2000). The effects of GABA on neural progenitors appear to be highly specific to the subunit composition of GABA receptors, and it remains to be elucidated how distinct subunit-containing receptors recruit different Rho GTPases. Interestingly, not only does GABAergic signaling recruit the Rho GTPases, it appears that Rho GTPases can also regulate components of the GABAergic postsynapse. The Cdc42-specific GEF Collybistin has been shown to regulate GABAergic neurotransmission as well as clustering of the main inhibitory postsynaptic scaffolding protein Gephyrin (Tyagarajan et al., 2011; Korber et al., 2012). This raises the interesting possibility that the small GTPases may also be playing a role in feedback or homeostatic mechanisms, influencing plasticity at the inhibitory postsynapse (Papadopoulos and Soykan, 2011). Collectively these results suggest that glutamatergic and GABAergic neurotransmission may be influencing newborn neuron maturation and spine development, at least in part by modulating small Rho GTPase activity.

### Developmental signaling molecules: notch

Notch signaling involves the activation of transmembrane heterodimer receptors, activated by Delta-like or Jagged, which are membrane-bound ligands present on neighboring cells. Ligand binding results in cleavage of the transmembrane domain of the notch receptor, releasing the notch intracellular domain (NICD), which translocates to the nucleus where it interacts with RBP-J to activate transcription of several genes. Notch signaling has been extensively explored during development and shown to be critical for neuronal cell-fate choice and neurite development during embryonic neurogenesis (Sestan et al., 1999; Redmond et al., 2000). Recently, studies have found Notch to also play important roles at different stages during adult neurogenesis (Ehm et al., 2010; Ables et al., 2011). In their study, using a conditional knockout of Notch1 in adult neural stem/progenitor cells, Breunig et al. show that in addition to playing a role in proliferation and differentiation, Notch1 is also important for dendritic arborization of newborn neurons (Breunig et al., 2007). Recent evidence indicates that non-canonical Notch signaling recruits the Trio-Rac1 (GEF-GTPase) complex for axon guidance in drosophila motor neurons, indicating that non-canonical Notch signaling may be specifically involved in neurite development via modulation of small Rho GTPase activity as compared to canonical Notch signaling (Song and Giniger, 2011). Although it has been hypothesized that Rho GTPase signaling likely mediates the effects of canonical Notch signaling on neurite growth, this remains to be confirmed (Redmond and Ghosh, 2001).

### Intermediate signal regulators

Recently, other intermediate signaling molecules such as Disrupted in schizophrenia 1 (DISC1) and Cyclin-dependent kinase 5 (Cdk5) were found to regulate the maturation of adult born neurons (Wu et al., 2013). In a hallmark study, Duan et al. found that cell-autonomous downregulation of DISC1 accelerated morphological maturation and aberrant integration of newborn neurons in the adult hippocampus. Their study revealed DISC1 to be a key regulator in maintaining the pace of maturation and integration of adult-born neurons (Duan et al., 2007). Following this study, several other groups found DISC1 to also play important roles in other aspects of neuronal maturation, such as axonal targeting, cell-cell/cell-matrix adhesion, and neurite outgrowth (Hattori et al., 2010; Kvajo et al., 2011). Interestingly, DISC1 has also recently been shown to be downstream of regulators previously shown to regulate neuronal maturation, for example, NMDARs (Namba et al., 2011; Wu et al., 2013). Despite the relatively recent discovery of DISC1's role in newborn neuron maturation, some studies have reported Rac1 to be specifically downstream of DISC1 in regulating neuronal maturation. A study in primary neurons *in vitro* found that DISC1 anchors the Rac1-GEF Kalirin-7 to the postsynaptic density, and regulates local Rac1 activity and spine morphology downstream of NMDAR activation (Hayashi-Takagi et al., 2010). Another study, using a heterologous DISC1 transgenic system in *C. elegans* motor neurons, found DISC1-mutant neurons to have abnormal axonal morphology, phenocopying Rac1-mutant defects. Further, it was found that DISC1 directly interacts with the Rac1 GEF, Trio, promoting Rac1 recruitment, suggesting that the observed axonal defects are likely due to impaired downstream Rac1 signaling (Chen et al., 2011). Additional studies are required to precisely characterize the role of small GTPases downstream of DISC1 in regulating neuronal maturation of adult-born hippocampal neurons.

Cdk5 has been shown to phosphorylate a number of neuronal proteins specifically involved in neuronal migration and synaptic plasticity (Jessberger et al., 2009; Lopes and Agostinho, 2011). A number of GEFs and small Rho GTPase effectors such as Ephexin1 (Fu et al., 2007), Ras Guanine nucleotide release factors 1 and 2 (Kesavapany et al., 2004, 2006), and Wave1 (Kim et al., 2006; Cheung and Ip, 2007) have been shown to be phosphorylated by Cdk5. In the context of adult hippocampal neurogenesis, Cdk5 appears to regulate adult neurogenesis, both in a cell-autonomous and non-cell-autonomous way. Retrovirus mediated overexpression of dominant negative Cdk5 leads to aberrant dendritic targeting and impaired spine maturation in a significant fraction of targeted adult-born hippocampal neurons, suggesting an intrinsic role for Cdk5 in neuronal maturation (Jessberger et al., 2008; Tobias et al., 2009). On the other hand, conditional deletion of Cdk5 in the NSPC population affected survival of immature neurons, an effect phenocopied in mice lacking the activating cofactor p35 (Lagace et al., 2008). This is particularly interesting because the p35/Cdk5 complex has been shown to be present at the growth cone, where it directly associates with Rac1, hyperphosphorylating its effector Pak1 kinase, resulting in an attenuation of Rac1 signaling (Nikolic et al., 1998). Interestingly, a recent study has placed Cdk5 downstream of BDNF in dendritic spines, *in vitro*, by showing that TrkB phosphorylation at specific serine residues (S478) is Cdk5 dependent. This phosphorylation of TrkB at S478 regulates its interaction with Rac1-GEF TIAM1, and downstream Rac1 activation (Lai et al., 2012). These results suggest that small Rho GTPases are downstream of Cdk5 signaling, and that Cdk5 may have distinct roles in adult neurogenesis via the differential recruitment of specific GEFs and GAPs. Taken together, data from these studies indicate strong links between DISC1, Cdk5, and Rac1 signaling in modulating neurite outgrowth dynamics *in vitro*, allowing us to speculate about such interactions *in vivo*.

## Mechanisms for signal convergence in adult-born hippocampal neurons

Research over the last two decades has given us a large amount of information regarding the molecular mediators downstream of diverse cellular inputs. We now understand how an upstream activator may have diverse effects on cellular behavior by recruiting multiple signaling cascades. In the context of maturation and integration of adult-born neurons, cellular behaviors such as neurite and spine growth are readouts, used as indicators of the state in which the cell is, as well as key endpoints with functional relevance. Neurite outgrowth and spine morphogenesis, at their core, are regulated by subcellular events leading to the stabilization, extension, or collapse of local cytoskeletal elements. Therefore, despite simultaneous activation of multiple signaling pathways, decisions of actual neurite growth or retraction are consequences of single subcellular events via modulation of local microtubules or actin filaments. *In vivo*, newborn neurons receive diverse input, each of which may simultaneously activate different pathways that can “instruct” neurite growth or neurite retraction. However, given the limited number of potential outcomes in neurite dynamics (i.e., either growth, stability, or collapse), we hypothesize that there must be mechanisms that allow for the convergence of multiple pathways leading to a single decision resulting in either neurite extension, stabilization, or collapse at a given time.

Studies exploring different signaling pathways have revealed how the bifurcation or divergence of signals via diverse signaling cascades enables different cellular responses to the same input, but it remains unclear how convergence of pathways might be taking place. One possible mechanism for signal convergence could be that following the peak of signal divergence some signaling cascades “funnel-in” by having fewer and fewer downstream targets until they directly influence cell neurite growth. For example, pathways such as mitogen-activated protein (MAP) kinase and CREB are common targets of many upstream regulators and have been hypothesized to act as signal integrators for certain cellular processes (Wagner and Nebreda, 2009; Merz et al., 2011). Given that an upstream input can recruit different signaling entities and the fact that there are limited context-specific cellular responses that can occur, there exists a dichotomy between high signal divergence initially followed by signal convergence, resulting in a single cellular event. Spatial and temporal segregation of signaling cascades obviously enables such divergence to take place. On the same note, spatial and temporal segregation can also facilitate convergence by promoting interaction of pathways within designated spatial and temporal contexts, for example at the post-synaptic density (Sheng and Kim, 2011). Similar concepts of signal convergence for pathways involving insulin, DAG/PKC, TGF-beta/BMP, G-proteins Gz/Gi have been previously discussed (Ho and Wong, 2001; Yang and Kazanietz, 2003; Taniguchi et al., 2006).

Central decision-making moieties are another mechanism through which signal convergence may occur. Among others, small Rho GTPases, due to their switch-like nature may serve to function as decision-making entities, for modulating neurite growth or collapse. As they are known to be downstream of a variety of activators, we further speculate that multiple upstream signals impinge upon the small Rho GTPases leading to decisive events shaping neurite and spine architecture. A way by which Rho GTPases may be assimilating upstream signal is by setting an activation (GTP-bound GTPase) threshold for upstream signaling. Further, regulating the concentration and regional availability of Rho GTPases may additionally enable localized assimilation of upstream signal. Sufficient input regulating the Rho GTPases' activity may come from multiple upstream pathways, where the Rho GTPases may be facilitating detection of coincident upstream input. Whether or not this is actually the case in maturation of newborn neurons remains to be resolved.

Clearly, the small Rho GTPases Cdc42, Rac1, and RhoA have functional ties with intrinsic regulators of neuronal maturation. Given their switch-like nature and a large repertoire of GEFs and GAPs regulating their state, it is likely that they are among the “decision-making” moieties via which diverse upstream regulators maybe decisively influencing neuronal architecture in the adult brain. So far, a majority of studies exploring the role of signaling molecules on neuronal function have utilized knockdown or overexpression models, *in vitro* and *in vivo*. While these approaches have generated valuable information about the basic roles of the Rho GTPases, their dispensability (knockout or dominant-negative constructs), and/or maximum functional capacity (overexpression or constitutively active constructs), we may have missed the nuanced roles of these signaling pathways of these signaling pathways under physiological conditions in neurons. Understanding whether such convergence indeed plays a role in signal transduction leading to decisive cellular events would minimally require experiments investigating activation of endogenous small GTPases, in real time, *in vivo*. This would provide us with significant insights into the physiological roles of these molecules and the decisive influence of regulatory pathways in mediating specific cellular behavior in the context of adult hippocampal neurogenesis.

### Conflict of interest statement

The authors declare that the research was conducted in the absence of any commercial or financial relationships that could be construed as a potential conflict of interest.
